# Chrysin Suppressed Inflammatory Responses and the Inducible Nitric Oxide Synthase Pathway after Spinal Cord Injury in Rats

**DOI:** 10.3390/ijms150712270

**Published:** 2014-07-10

**Authors:** Yong Jiang, Fu-Liang Gong, Guang-Ben Zhao, Jie Li

**Affiliations:** Orthopedics Department, the First Affiliated Hospital of Dalian Medical University, 222 Zhong Shan Road, Dalian 116011, China; E-Mails: yongjiangdr2014@163.com (Y.J.); guangbenzhaodr2014@163.com (G.-B.Z.); jielidr2014@163.com (J.L.)

**Keywords:** chrysin, spinal cord injury, neuroprotection, inflammation, inducible nitric oxide synthase

## Abstract

Chrysin (CH), a natural plant flavonoid, has shown a variety of beneficial effects. Our present study was conducted to evaluate the therapeutic potential of CH three days after spinal cord injury (SCI) in rats and to probe the underlying neuroprotective mechanisms. SCI was induced using the modified weight-drop method in Wistar rats. Then, they were treated with saline or CH by doses of 30 and 100 mg/kg for 26 days. Neuronal function was assessed with the Basso Beattle Bresnahan locomotor rating scale (BBB). The water content of spinal cord was determined after traumatic SCI. The NF-κB p65 unit, TNF-α, IL-1β and IL-6 in serums, as well as the apoptotic marker, caspase-3, of spinal cord tissues were measured using commercial kits. The protein level and activity of inducible nitric oxide synthase (iNOS) were detected by western blot and a commercial kit, respectively. NO (nitric oxide) production was evaluated by the determination of nitrite concentration. The rats with SCI showed marked reductions in BBB scores, coupled with increases in the water content of spinal cord, the NF-κB p65 unit, TNF-α, IL-1β, IL-6, iNOS, NO production and caspase-3. However, a CH supplement dramatically promoted the recovery of neuronal function and suppressed the inflammatory factors, as well as the iNOS pathway in rats with SCI. Our findings disclose that CH improved neural function after SCI in rats, which might be linked with suppressing inflammation and the iNOS pathway.

## 1. Introduction

It is well established that spinal cord injury (SCI) serves as a devastating health problem that affects thousands of individuals [1] each year and causes primary and secondary injuries [2]. The primary trauma causes mechanical compression, bleeding and electrolyte disturbance, finally resulting in irreversible nerve injury. Meanwhile, the delayed secondary impairment is made up of multiple pathophysiological processes, including ischemia, edema, hemorrhage, inflammatory responses, energy metabolism system disorder, excitotoxicity and oxidative damage [3], which generates reversible nerve injury. Besides, the secondary lesion could be modulated, and this has been considered to be a critical step for treating SCI [4,5]. In the meantime, several inflammatory factors could influence the growth and survival of cells, finally facilitating neuronal functioning following traumatic SCI [6]. Evidence supports that glucocorticoids, the endogenous anti-inflammatory steroids, are reported to regulate inflammatory reactions in response to SCI [6].

Nitric oxide (NO) is one of the major participants in secondary damage after SCI. Additionally, NO could cause marked inflammation in the regulation of immune responses [7]. It is well established that NO is synthesized by two classes of enzyme proteins, including Ca^2+^-dependent constitutive nitric oxide synthase (cNOS) and Ca^2+^-independent inducible nitric oxide synthase (iNOS). Emerging evidence has illustrated that inflammation-induced iNOS is highly expressed, and this isoform produces great amounts of NO after traumatic SCI, consequently causing cytotoxicity to spinal cord [8,9]. Additionally, it was also previously reported that NO synthesized by iNOS triggered marked cellular apoptosis after SCI [10], suggesting that inhibition of this isoform could be beneficial for reversing secondary damage after SCI in the clinic.

Chrysin (CH), an important natural plant flavonoid, has been considered to possess multiple biological effects, including antioxidative, anti-inflammatory and antiapoptotic properties in the central nervous and immune systems [11,12,13]. Recently, CH was reported to effectively alleviate oxidative insults and apoptosis in primary rat mesencephalic cultures [13]. Besides, CH could also exert a protective role against brain damage caused by chronic cerebral hypoperfusion in rats [14]. However, there are no studies regarding the therapeutic potential of CH for SCI. Furthermore, as inflammation and the iNOS pathway play a critical role in the pathogenesis of SCI, we speculate that they are involved in CH’s neuroprotection. The present investigation aimed to evaluate the protection of CH and elucidate the roles of inflammation and the iNOS pathway in CH-mediated neuroprotection against traumatic SCI in rats.

## 2. Results and Disscussion

### 2.1. CH (Chrysin) Improves the Recovery of Neurological Function and Diminishes the Water Content of Spinal Cord after SCI (Spinal Cord Injury)

**Figure 1:**
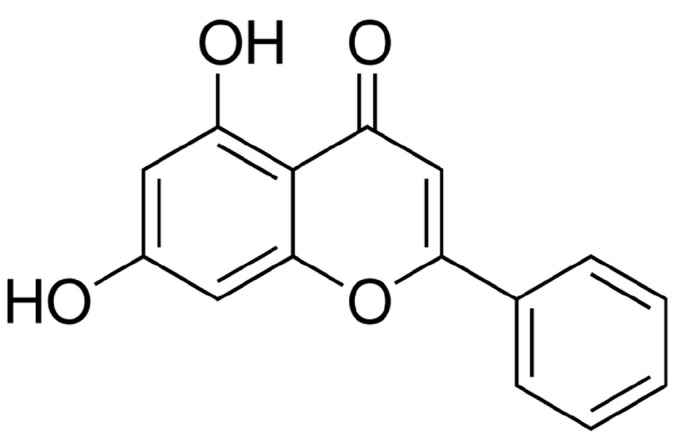
The chemical structure of chrysin.

The chemical structure of CH is displayed in Figure 1. Table 1 lists Basso Beattle Bresnahan (BBB) scores in different groups. The rats induced by SCI exhibited a severe impairment with remarkable reductions of the BBB scores (*p* < 0.01) at the selected time points. However, treatment with CH at different doses (30 and 100 mg/kg) to injured rats significantly improved the neurological function (*p* < 0.01), in comparison to the SCI model group.

**Table 1:** BBB (Basso Beattle Bresnahan) scores for rats at 24, 48 and 72 h after SCI (spinal cord injury). MPSS, methylprednisolone; CH, chrysin.

Animal Groups	*n*	24 h	48 h	72 h
Sham	10	16.54 ± 0.87	17.24 ± 1.12	19.18 ± 1.32
SCI	10	4.15 ± 0.67 **	3.87 ± 0.92 **	4.12 ±0.33 **
MPSS	10	12.83 ± 0.55 ^##^	13.12 ± 0.87 ^##^	14.21 ± 0.66 ^##^
CH (30 mg/kg)	10	8.79 ± 0.35 ^##^	9.12 ± 0.66 ^##^	10.05 ± 0.86 ^##^
CH (100 mg/kg)	10	9.05 ± 0.64 ^##^	9.85 ± 0.83 ^##^	10.11 ± 1.22 ^##^

** *p* < 0.01 *versus* the sham group; ^##^ *p* < 0.01 *versus* the SCI group.

**Figure 2:**
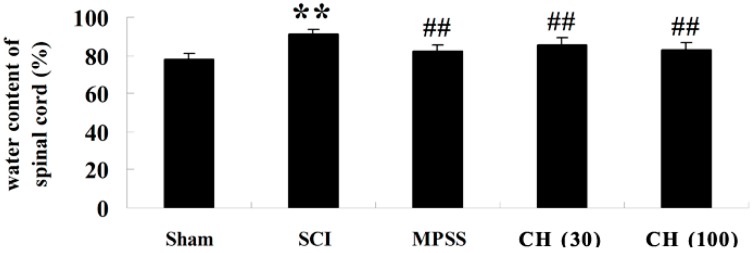
CH diminishes the water content of spinal cord after SCI (*n* = 10, mean ± SD). ** *p* < 0.01 compared with the sham group; ^##^ *p* < 0.01 compared with the SCI group. Sham, sham group; SCI, spinal cord injury group; MPSS, methylprednisolone-treated; CH (30), chrysin (30 mg/kg)-treated; CH (100), chrysin (100 mg/kg)-treated groups.

Besides, it was noted that the injured rats had extremely high water content of spinal cord (*p* < 0.01). However, this phenomenon was significantly reversed by CH treatment at the doses of 30 and 100 mg/kg (*p* < 0.01), as shown in Figure 2.

### 2.2. CH Suppresses Inflammation after SCI

**Figure 3:**
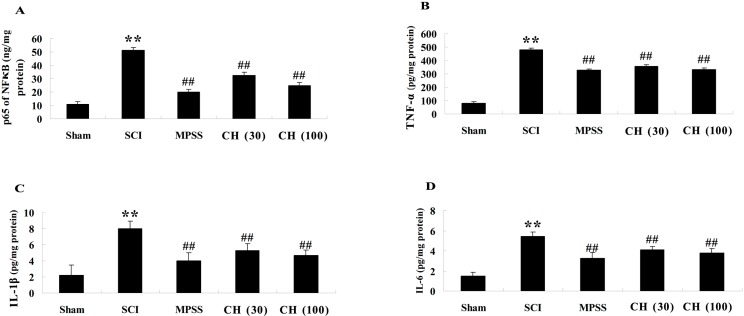
CH inhibits the levels of the NF κB p65 subunit (**A**); TNF-α (**B**); IL-1β (**C**) and IL-6 (**D**) after SCI (*n* = 10, mean ± SD). ** *p* < 0.01 compared with the sham group; ^##^ *p* < 0.01 compared with the SCI group. Sham, sham group; SCI, spinal cord injury group; MPSS, methylprednisolone-treated; CH (30), chrysin (30 mg/kg)-treated; CH (100), chrysin (100 mg/kg)-treated groups.

It was previously reported that the serum cytokine levels were more relevant to SCI [15]. Therefore, the serum levels of inflammatory cytokines were determined in our current investigations. As shown in Figure 3A–D, the NF-κB p65 unit, TNF-α, IL-1β and IL-6 in serums were all significantly elevated three days post SCI injury (*p* < 0.01), and CH treatment of SCI-induced rats markedly suppressed these indices (*p* < 0.01). The anti-inflammatory effect of 100 mg/kg CH was equipotent to MPSS (methylprednisolone) (*p* > 0.05).

### 2.3. CH Decreases the Protein Expression and Activity of iNOS (Inducible Nitric Oxide Synthase) and Plasma NO (Nitric Oxide) Concentration after SCI

**Figure 4:**
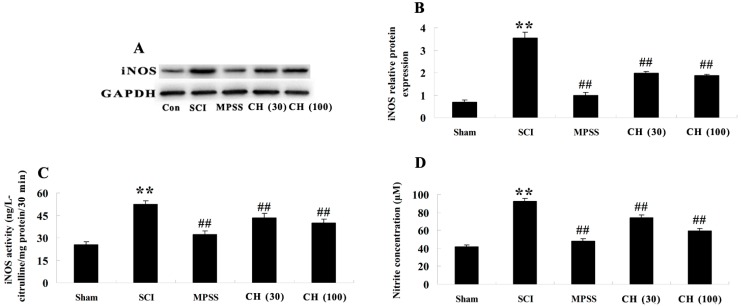
CH decreases the protein level and activity of iNOS (inducible nitric oxide synthase) and the plasma NO (nitric oxide) concentration after SCI. (**A**) The representative images of immunoblots with antibodies against iNOS. iNOS, 130 kDa; GAPDH, 36 kDa; (**B**) The quantitative analysis of the protein level of iNOS in rats’ spinal cords from different groups. The data were normalized to the loading control, GAPDH; (**C**) The measurement of iNOS activity; (**D**) NO production was detected spectrophotometrically by measuring its metabolite, nitrite. ** *p* < 0.01 compared with the sham group; ^##^ *p* < 0.01 compared with the SCI group. Sham, sham group; SCI, spinal cord injury group; MPSS, methylprednisolone-treated; CH (30), chrysin (30 mg/kg)-treated; CH (100), chrysin (100 mg/kg)-treated groups.

We further investigate whether CH exerted protection against SCI through mediation of iNOS. Figure 4A revealed that the western blot with iNOS antibody showed the anticipated bands of 130 kDa. Quantitative analysis disclosed an evident elevation of iNOS protein in SCI-subjected rats (*p* < 0.01), compared to the control group (Figure 4B). Nevertheless, CH treatment (30 and 100 mg/kg) remarkably decreased the protein expression of iNOS in the SCI group (*p* < 0.01), compared with the vehicle. In order to verify the alteration of iNOS after SCI, iNOS activity was further measured using commercial kits. Figure 4C revealed an obvious elevation of iNOS activity in injured rats, and CH inhibited this value by the selected doses (*p* < 0.01). Taken together, the NOS activity we see corresponded to iNOS based on the western blot data and biological consistency. Additionally, NO production, which was indicated as nitrite formation, was found to be significantly increased after SCI (*p* < 0.01). CH induced a remarkable reduction in the nitrite level (*p* < 0.01) (Figure 4D).

### 2.4. CH Inhibits the Caspase-3 Activity after SCI

**Figure 5:**
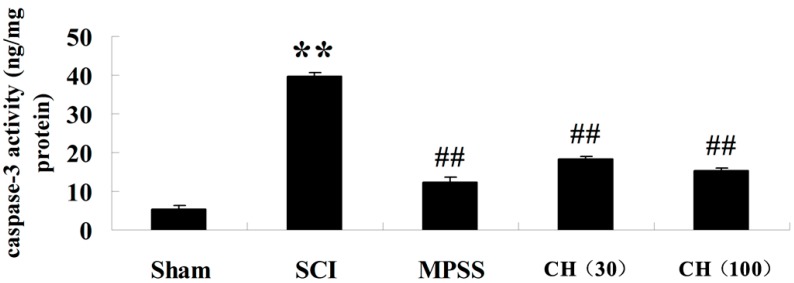
CH suppresses caspase-3 activity in spinal cord tissues after SCI (*n* = 10, mean ± SD). ** *p* < 0.01 compared with the sham group; ^##^ *p* < 0.01 compared with the SCI group. Sham, sham group; SCI, spinal cord injury group; MPSS, methylprednisolone-treated; CH (30), chrysin (30 mg/kg)-treated; CH (100), chrysin (100 mg/kg)-treated groups.

Figure 5 revealed that caspase-3 levels were remarkably augmented in spinal cord tissues of the SCI model group (*p* < 0.01). However, administration with CH (30 and 100 mg/kg) generated a more pronounced reduction of caspase-3 activity in injured animals (*p* < 0.01). Moreover, the anti-apoptotic effect of CH at a dose of 100 mg/kg was equipotent to that of MPSS.

### 2.5. Discussion

Methylprednisolone (MPSS) is a well-accepted antioxidant-anti-inflammatory agent that alleviates secondary injuries after SCI. In our present investigation, we compared the effect of MPSS and CH treatment in an acute SCI rat model. A high dose of MPSS was found to inhibit oxidative stress and improved neurological function after traumatic SCI [16]. The major findings of our study illustrated that treatment with CH protected against neuronal damage after SCI, and its neuroprotection might be associated with suppressing inflammatory responses and iNOS signaling.

The BBB score is a very common method for evaluating edema in the spinal cord [16]. It is widely used in detecting the neuroprotective effect of many drugs against spinal cord edema [17,18]. It was previously reported that SCI caused a low BBB score, and salvianolic acid B treatment significantly improved BBB scores in rats with SCI [18]. Consistently, the BBB score was obviously decreased in the SCI group at different time points, and it was evidently increased in the CH-treated group. These findings suggest that the use of CH improved the locomotor function in rats after SCI.

Inflammation is a crucial factor in secondary damage after SCI [19]. It was reported that SCI could trigger the increased levels of serum TNF-α and IL-1β [20]. Additionally, the therapeutic reagents that diminish either TNF-α or IL-1β production have been found in other models of neurological diseases, including ischemia, traumatic brain injury and SCI [21]. In our present study, the activities of major inflammatory cytokines, including the NF-κB p65 subunit, TNF-α, IL-1β and IL-6, were all remarkably augmented in SCI-induced rats, and CH treatment dose-dependently suppressed the proinflammatory responses, which implicates the involvement of its anti-inflammatory action in the attenuation of SCI in rats.

It was reported that inflammatory reactions could cause the increased expression of iNOS, leading to the excessive NO production after SCI [8]. The high amounts of NO have a cytotoxic effect on spinal cords. In fact, selective inhibition of iNOS by aminoguanidine was found to improve the recovery of neurological function in rats subjected to SCI [22]. Consistent with part of the previous results, our current work depicted that the protein level and activity of iNOS together with NO production were all evidently increased in injured rats. Collectively, the NOS activity we see corresponded to iNOS based on the western blot data and biological consistency. Additionally, what is more important, the administration of CH dramatically reduced these indices in the traumatic SCI rat model. These findings imply that CH exerted protective effects against spinal cord impairment via suppressing iNOS and concomitantly decreasing NO bioavailability.

The activations of inflammatory responses and the iNOS pathway both damage cells and induce apoptotic cascades during traumatic SCI. The pharmacological inhibition of apoptosis may serve as a promising therapeutic strategy. The administration of an anti-apoptotic agent after SCI may help rescue partially damaged cells in the penumbra before they make the commitment to apoptosis, thereby restoring neurological function [23]. Caspase-3 is regarded as an executioner molecule in the apoptotic cascades. Results from our current work depicted that there was a dramatic elevation of caspase-3 in rats after SCI. However, the administration of CH significantly inhibited the activity of caspase-3, and the inhibitory effect by a dose of 100 mg/kg was equipotent to the MPSS group. What is more, it was noted that treatment with CH by a dose of 100 mg/kg was more effective against various indices than that of 30 mg/kg, with no obvious adverse effects (data not shown) in our study, suggesting that 100 mg/kg of CH may be a more efficient dose for treating SCI. Further investigations will be required to justify this conclusion.

## 3. Experimental Section

### 3.1. Animals

Adult Wistar rats weighing about 230–250 g were selected in our present investigation. The rats were supplied from Beijing Animal Center. They were maintained under a controlled environment with a 12:12 h day/night cycle and 50%–70% humidity. Free access to water and food were provided for these animals. Great efforts were made to minimize the number of animal used and their suffering. All surgical procedures throughout our study were conducted according to the guidelines of the Care and Use of Laboratory Animals of the first affiliated hospital of Dalian Medical University.

### 3.2. Drugs and Chemicals

CH (with a purity >98% by HPLC analysis) was purchased from Nanjing Tcm Institute of Chinese Material Medica (Nanjing, China). MPSS was supplied by Nanfang Hospital of Guangzhou in China (Pharmacia & Uprohn, Excenel, Belgium). TNF-α, IL-1β and IL-6 ELISA kits were supplied by R&D Systems (BiosPacific, CA, USA). The NF κB p65 unit ELISA kit was bought from Imgenex (San Diego, CA, USA). All other reagents were of analytical grade.

### 3.3. Induction of SCI Rat Model and Drug Treatment

The rat model of spinal cord injury was induced as previously described [24]. Briefly, after anesthesia with pentobarbital (50 mg/kg, i.p.), the skin of rats above the vertebral column was shaved carefully and cleaned using betadine solution. Thereafter, a 20-mm midline incision was made in the thoracic region in order to exposing the vertebral column. Finally, we carried out the laminectomy at vertebral level T-10, and the dorsal cord surface was exposed with the dura remaining intact. The injury was generated by dropping a 10-g rod from a height of 5.0 cm onto the spinal cord at T-10. Animals in each experiment were randomly divided into four groups: (1) sham group (Sham) (*n* = 10), which exposed the operational area, but had no trauma hit (physiological saline 0.1 mL/100 g, i.p.); (2) spinal cord injury group (SCI) (*n* = 10), which experienced spinal cord injuries and received saline (physiological saline 0.1 mL/100 g, i.p.); (3) MPSS group (*n* = 10), which was treated with 100 mg/kg of MPSS (i.p.); (4–5) CH groups (CH30 and CH100) (*n* = 10), which had spinal cord injuries and were treated with CH at doses of 30 and 100 mg/kg once a day for 26 consecutive days, respectively. The dosage and dosing frequency of CH was chosen according to the previous report [14].

### 3.4. Evaluation of Neuronal Function Recovery

The evaluation of locomotor recovery after SCI was conducted by the Basso Beattie Bresnahan (BBB) locomotor rating scale of 0 (complete paralysis) to 21 (normal locomotion) [25]. BBB scores contain combinations of rat hindlimb movements, trunk position and stability, stepping, coordination, paw placement, toe clearance and tail position, representing the sequential recovery stages that rats acquire after spinal cord injury.

### 3.5. Assessment of Water Content in Spinal Cord Tissue

Spinal cord edema was assessed by calculating the water content of the spinal cord. Briefly, 72 h after SCI, the injured spinal cords were dried for 48 h at −80 °C for the measurement of dry weight. Water content in spinal cords was calculated by the following formula: water content of spinal cord (%) = (wet weight − dry weight)/wet weight × 100%.

### 3.6. Measurement of Serum NF-κB p65 Unit, TNF-α, IL-1β and IL-6 Levels

The activities of the serum NF-κB p65 unit, TNF-α, IL-1β and IL-6 were analyzed by the respective commercial immunoassay kits according to the manufacturers’ protocols (R&D Systems).

### 3.7. Western Blot Analysis

After treatment with CH for 26 consecutive days, the injured spinal cord tissues were homogenized in an ice-cold lysis buffer. The supernatant was collected after centrifugation at 12,000× *g* for 20 min and protein quantification was conducted by a BCA kit (Beyotime Institute of Biotechnology, Nantong, China). Sixty micrograms of protein were separated by electrophoresis on 8% or 10% SDS-polyacrylamide gels and transferred onto nitrocellulose membranes. Protein was detected using mouse anti-iNOS (1:300, Santa Cruz, Dallas, TX, USA) or mouse anti-GAPDH (1:2000, Kang Chen, Shanghai, China) and horseradish peroxidase-conjugated goat antimouse antibody (1:5000, Santa Cruz). Quantitative analysis was conducted by Quantity One software (Bio-Rad, Hercules, CA, USA).

### 3.8. Determination of iNOS Activity

Spinal cord tissues were collected 72 h after SCI from different groups. iNOS activity was determined using commercial kits according to the manufacturer’s instructions (Nanjing Jiancheng Biotechnology Institute, Nanjing, China).

### 3.9. Assay of Plasma NO Production

Seventy two hours after traumatic SCI, the plasma supernatant was collected after centrifugation at 2000× *g* for 20 min at 4 °C. The nitrite concentration was spectrophotometrically determined using Griess reagent (1% sulfanilamide and 0.1% naphthylethylenediamide in 5% phosphoric acid).

### 3.10. Measurement of Caspase-3 Activity in Spinal Cord Tissues

As an executioner molecule in the apoptotic cascades, caspase-3 was determined by the cleavage of chromogenic caspase substrates, Ac-DEVD-pNA, a protease that is rapidly activated when cells are exposed to apoptotic conditions and that cleaves poly(ADP-ribose) polymerase. The amount of caspase-3 was read at a wavelength of 405 nm by spectrophotometer according to the manufacturer’s instructions (R&D Systems, BiosPacific, CA, USA).

### 3.11. Statistical Analysis

All of the data were expressed as the mean ± SD and analyzed using one-way ANOVA followed by Dunnett’s test. A value of *p* < 0.05 was regarded as statistically significant.

## 4. Conclusions

In conclusion, our current work firstly depicted that CH treatment promotes neurological recovery after SCI and its neuroprotection is, at least, partly associated with suppressing inflammatory reactions and the iNOS pathway.
